# Are we meeting the calorific and protein requirement needed in the intensive care setting?

**DOI:** 10.1186/2197-425X-3-S1-A579

**Published:** 2015-10-01

**Authors:** ST Passey, AKA Abi Musa Asa'ari, M Mendis, B Carr

**Affiliations:** Critical Care Unit, University Hospitals of North Midlands Trust, Stoke-on-Trent, United Kingdom

## Introduction

Establishing nutrition support in a timely manner and by the appropriate route is a priority in the management of the acutely unwell patient. We investigated the total calorific and protein input received in a cohort of patients in the first 7 days of nutrition support during their critical care stay.

## Objectives

We examined the current practice of our intensive care unit (ICU) regarding the amount of calories and protein given to patients during the first days of their critical care stay to assess achievement of energy and protein delivery with respect to consensus guidelines.

## Methods

We collected retrospective data from 50 consecutive intensive care patients from July to November 2014. The inclusion criterion was patient's receiving ≥7 days of prescribed enteral/parenteral feed. Data collected included; patient demographics, type and amount of feed given and total propofol administered in the same period. 3 patients were excluded due to no weight recorded.

We calculated the calorie and protein goal per patient for the 7 day period according to guidelines. [1, 2] We then worked out each patient total intake and expressed that as a percentage of the goal target; therefore comparing our practice to the standards. The calories calculated included those provided in propofol.

ASPEN guidelines suggest delivery of >50 to 65% of goal calories (GC) in their first week. GC in the acute phase is 25 kcal/kg/day. For patients with BMI of < 30, GC was calculated from actual body weight whereas with BMI >30, GC was calculated from ideal body weight. For protein intake ESPEN and ASPEN both recommend 1.5 g/kg/day for BMI < 30.2 g for 30-40, 2.5 g for >40.

## Results

87.2% of patients received >65% of their weekly GC and 8.5% received between 50 to 65%.

In the first 7 days from commencement of feed. Only 1 patient (2.1%) received the required amount of protein according to his BMI. 83% received between 50 and 99% of the required total.

60% of patients were initially prescribed a high protein feed as per local guidelines. 26% of patients received low protein feed contrary to local guideline.

## Conclusions

66% of the patients were started on feed within 48 hours of admission. 95.7% of our patients were receiving adequate calorific intake according to guidance. [1, 2]

Provision of protein to our critically ill patients remained low despite predominant use of high protein feed.

**Figure 1:**
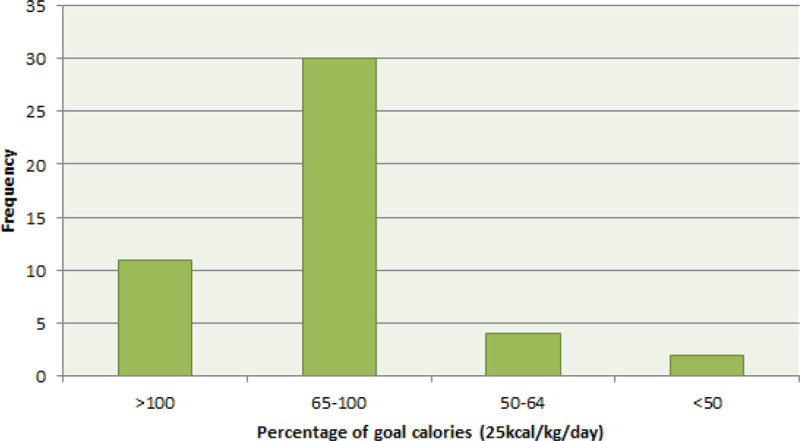
**Frequency of percentage goal calories in 7 days.**

**Figure 2:**
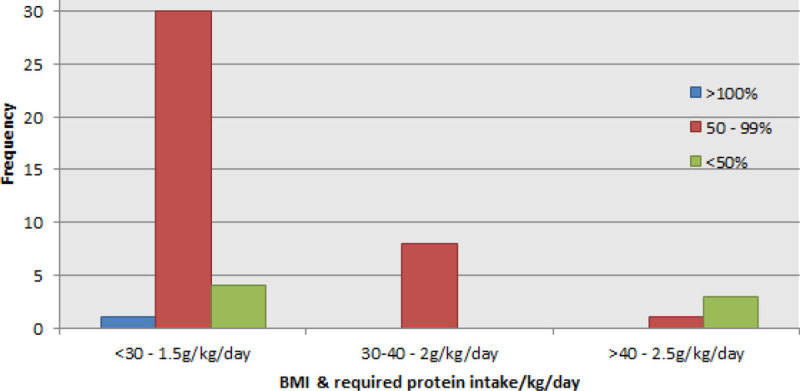
**Frequency of percentage goal protein in 7 days according to BMI.**

**Figure 3:**
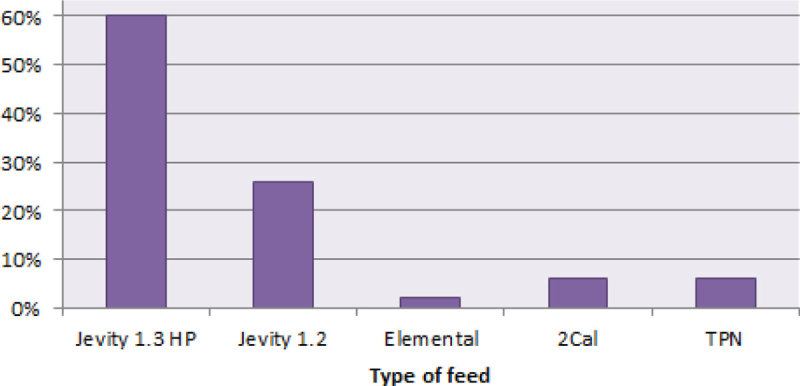
**First feed initiated post admission.**
